# Use of a Telerehabilitation Platform in a Stroke Continuum: A Qualitative Study of Patient and Therapist Acceptability

**DOI:** 10.5195/ijt.2022.6453

**Published:** 2022-12-13

**Authors:** Isabelle Gaboury, Rosalie Dostie, Hélène Corriveau, Arnaud Demoustier, Michel Tousignant

**Affiliations:** 1 Department of Family Medicine and Emergency Medicine, Université de Sherbrooke, Longueuil, Québec, Canada; 2 School of Rehabilitation, Université de Sherbrooke, Sherbrooke, Québec, Canada; 3 School of Nursing, Université de Sherbrooke, Longueuil, Québec, Canada

**Keywords:** Acceptability, Healthcare professionals, Multidisciplinary team, Qualitative interview, Stroke, Telerehabilitation

## Abstract

The purpose of this study was to describe the acceptability of a stroke telerehabilitation platform from the perspective of both patients and therapists. Two public rehabilitation centers participated in a pilot telerehabilitation trial. A theoretical framework was used to conceptualize acceptability. Semi-structured individual interviews with patients and focus groups of therapists were conducted. Most participants and therapists were satisfied with the intervention. Participants emphasized the advantages of staying at home to get their treatments. Therapists were more skeptical at first about their self-efficacy to deliver therapy remotely. There was a consensus among therapists about the need for a combination of telerehabilitation and in-person visits to optimize treatments. While we found overall good acceptability, effectiveness of this technology could be improved via an accessible user interface, complementary rehabilitation material, and ongoing training and technical just-in-time support with therapists.

In Canada, stroke is the leading cause of adulthood disability and is associated with substantial morbidity and mortality (Virani et al., 2020). Three-quarters of survivors of stroke will live with minor to severe impairments or disabilities (Krueger et al., 2015). Returning home shortly after a stroke event places the patient in the most favourable environment to foster the success of rehabilitation therapy (Anderson et al., 2000; Fisher et al., 2011; Richards, 2013). A major issue emerging from the implementation of this recommendation (Campbell, 2012) is the requirement for some patients to travel long distances to access outpatient rehabilitation services. As a result, many patients refuse their rehabilitation treatments outright or decrease their adherence to treatments over time, which have been shown to impact patient health negatively (GTA Rehab Network, 2011).

Telerehabilitation, which refers to the use of technology to provide long-distance rehabilitative services (OPPQ, 2018; Georgeadis et al., 2004; Kairy et al., 2009; Ward et al., 2009), is recommended by the Canadian Stroke Guidelines to ensure equal and timely access to optimal stroke services (Blacquiere et al., 2017; Boulanger et al., 2018). Telerehabilitation for patients who have survived stroke have been documented and evidence towards its efficacy and efficiency has emerged (Dodakian et al., 2017; Grant et al., 2002; Holden et al., 2007; Lai et al., 2004; Lin et al., 2014; Sarfo et al., 2018). To ensure broad use of this technology, acceptability of this innovation needs to be explored more thoroughly (Borrelli et al., 2005; Proctor et al., 2009).

Acceptability is a key concept when it comes to successfully implementing any healthcare intervention. Acceptability can be theoretically defined as a multifactorial construct expressing the extent to which patients or therapists consider an intervention to be appropriate according to their cognitive or emotional expectations with regards to the intervention (Sekhon et al., 2017). Sekhon et al., in a meta-synthesis of the concept of acceptability, operationalize it through seven components (Table 1).

**Table 1:** Seven Components of Acceptability According to Sekhon et al., 2017

Affective attitude	Feelings of the patients about the intervention
Burden	Effort needed to participate
Perceived effectiveness	User's perception of the capacity of the intervention to live up to its purpose
Ethicality	Fit between the user's values and the intervention
Intervention coherence	Understanding of the intervention from the user's point of view
Opportunity costs	Direct or indirect cost to engage in the intervention
Self-efficacy	User's level of confidence to engage in the intervention

Only a few studies have looked at the acceptability of a telerehabilitation intervention for persons with stroke (Brouns et al., 2018; Davoody & Hagglund, 2016; Macko et al., 2016; Tyagi et al., 2018; Vourganas et al., 2019). So far, acceptability has been studied mostly using visual analogue scales (Macko et al., 2016) or questionnaires (Kringle et al., 2020; Lai et al., 2004). However, most have operationalized the concept through feasibility (Beit Yosef et al., 2019; Held et al., 2018; Isernia et al., 2019; Johansson & Wild, 2011), satisfaction (Beit Yosef et al., 2019; Benvenuti et al., 2014; Brouns et al., 2018; Chen et al., 2020; Crotty et al., 2014; Johansson & Wild, 2011) or adherence (Benvenuti et al., 2014).

Overall, most persons with stroke evaluate telerehabilitation as being helpful for their recovery, with very high levels of satisfaction (Beit Yosef et al., 2019; Chen et al., 2020; Crotty et al., 2014; Lai et al., 2004; Macko et al., 2016). However, some report concerns about their capability to use the platform (Brouns et al., 2018; Tyagi et al., 2019; Tyagi et al., 2018), and fatigue (Brouns et al., 2018). Authors agree that to achieve higher acceptability, telerehabilitation platforms should be customized to meet patients' needs, be appropriate for elderly patients (i.e., user friendly), consider user's tolerance to avoid fatigue (Brouns et al., 2018) and limit the use of wearable add-ons or intrusive monitoring (Vourganas et al., 2019).

Most therapists claim that telerehabilitation could support the patients in their treatment (Davoody & Hagglund, 2016); however, therapists' principal concern was the requirement for a certain level of familiarity with technology (Davoody & Hagglund, 2016). Therapists also believed they would not have sufficient time to use a telerehabilitation platform in their day-to-day job (Brouns et al., 2018; Davoody & Hagglund, 2016; Tyagi et al., 2018). Unfortunately, therapists interviewed in two of these studies had never experienced telerehabilitation; studies have reported on a hypothetical platform (Brouns et al., 2018; Davoody & Hagglund, 2016). Evaluation of acceptability, from a therapist's perspective, has thus never been thoroughly conducted.

This study aims to describe the acceptability of a telerehabilitation platform for stroke rehabilitation from the perspective of both patients and therapists and to propose recommendations to ease its implementation.

## Methods

To get a comprehensive understanding of the phenomenon under study (i.e., acceptability of the platform) (Sandelowski, 2000), a qualitative descriptive study design was adopted (Kim et al., 2017). Qualitative interviews were conducted as part of a before-after pilot trial of a telerehabilitation platform within a stroke rehabilitation continuum.

### Intervention

**Figure 1:**
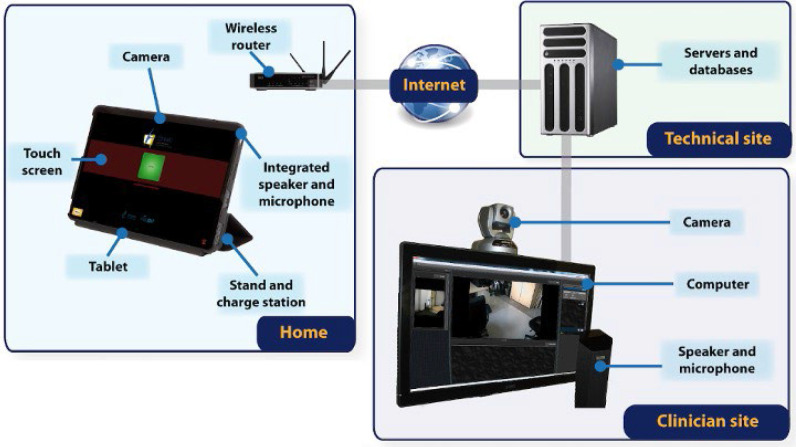
Schematic Flowchart of the Telerehabilitation Platform

The platform, OpenTera, is a cloud-based multi-point, multi-view and multi-stream (video and audio) telecommunication system with proven usability and robustness for telerehabilitation applications (Figure 1). The platform is not linked to a specific commercial platform or to special network configurations. An optical zoom is available for specific views when required. A second camera (document camera) is installed for both clinician and patient close-ups of certain body parts (e.g., hands for writing exercises).

Patients were recruited for the pilot study immediately following acute care (stroke unit) or following in-patient rehabilitation. Patients were eligible if they were male or female adults (18+ years) with a stroke event (ischemic or hemorrhagic) and: (a) had a modified Rankin score between 1 and 3 (Quinn et al., 2009); (b) had a spouse or informal caregiver who could attend physical rehabilitation treatments; and (c) had either access to high-speed Internet or accepted to have it installed in their home for the duration of the trial. Patients with severe cognitive decline prior to the trial or aphasia were excluded. Patients were lent a tablet to access the platform for the duration of their rehabilitation treatments. When necessary, an Internet connection was provided, or signals boosted to allow good reception of audio and video.

Rehabilitation teams from two public rehabilitation centers in Quebec, Canada, were recruited for the study. Teams were instructed to plan rehabilitation care as they would do for non-virtual therapy, while delivery could be modified (e.g., accessories used, frequency and dose). Rehabilitation techniques focused on traditional physical therapy exercises, including neuromuscular facilitation which involves sensorimotor stimulation (Díaz-Arribas et al., 2020) and task-specific approach designed to enhance motor recovery (Rensink et al., 2009). Specific cognitive rehabilitation and language therapy followed recent practice guidelines (Heart & Stroke Foundation, 2018; Mountain et al., 2020; Teasell et al., 2020) based on the patient's condition and needs. Each team had access to the telerehabilitation platform in a dedicated room. Therapists, including physiotherapists, occupational therapists, social workers, speech-language pathologists, nutritionists, and neuropsychologists were trained by the research team to use the platform. Two 2-hour interactive workshops were offered to: (1) get familiar with the technology; and (2) learn how to adapt stroke rehabilitation exercises through a telerehabilitation platform. Ongoing training was offered on a need basis. Therapists were instructed to refrain from delivering in-person care to the participants, unless their safety was at stake.

### Data Collection

Sekhon's conceptual framework of acceptability (Sekhon et al., 2017) was used to develop two semi-structured interview guides (patients and therapists, Appendix). Patients were interviewed individually by telephone at the end of the intervention (ranged 2 to 16 weeks). Therapists met in focus groups (one for each site) when all patients had ended their follow-up.

### Data Analysis

Individual and group interviews were audiotaped and then transcribed verbatim. Transcripts were coded and analyzed using the NVivo software. Using a hybrid inductive and deductive approach (Fereday & Muir-Cochrane, 2006), a research assistant (RD) generated a coding system based on the study's conceptual framework. The coding, themes, and subthemes were discussed frequently with the principal investigator. Through this process emerged the final themes (Braun & Clarke, 2006). Matrix coding was also used to compare patients' and therapists' related themes (Miles et al., 2020). To enhance trustworthiness, member checking was conducted with the therapists from both teams. Ethics approval has been granted by the two local Research ethics committees.

## Results

All participants were invited for a qualitative interview. See Table 2 for sociodemographic characteristics as well as participant clinical profiles based on selected measures. All patients recruited had suffered an ischemic stroke. Interviews were conducted with 5 of the 13 eligible participants (patients 1–5 in Table 2). Other participants refused to participate because they were not available, showed signs of fatigue, or for personal reasons. Focus groups were conducted with 21 rehabilitation team members. The interviews took place between March and August 2018.

**Table 2:** Patient Characteristics

Sociodemographics													
	1	2	3	4	5	6	7	8	9	10	11	12	13
Age	73	84	71	68	71	77	68	45	80	77	52	63	54
Gender	M	M	F	M	M	M	M	F	M	M	M	F	F
Time since stroke (days)	8	7	6	8	8	12	79	77	6	10	16	45	150
Intervention length (weeks)	12	11	9	13	13	16	19	13	10	10	14	LFU	LFU
Services received^*^	Pt, OT, SLP, SW	OT	OT, SLP, N	OT, SLP	Pt, OT	OT, SLP, Pt	OT, Pt, SEd	OT, SLP, Pt, Neuro	OT, SLP, Pt, Neuro, SW	OT, SPL, Pt, SW	OT, SW, Neuro	OT, Pt, SW, SEd	OT, SLP
Hemisphere affected^*^	R	–	–	–	–	L	L	R-L	R-L	–	R	R-L	L
Pre-intervention													
Modified Rankin score (Quinn et al., 2009)	2	2	3	1	1	1	1	2	2	2	1	2	2
Reintegration to normal living index (/22)^**^ (Wood-Dauphinee & Williams, 1987)	22	15	17	15	22	21	13	12	15	15	19	22	14
Beck depression inventory (short form)+ (Beck et al., 1974)	1	3	9	2	1	6	8	10	12	14	2	0	23
Quality of life (EQ-5D) (/100)(Oppe et al., 2007)	80	50	80	60	85	70	50	80	65	70	80	90	50
Post-intervention													
Modified Rankinscore (Quinn et al., 2009)	2	1	3	1	1	1	1	2	2	1	1	–	–
Reintegration to normal living index (/22)^**^ (Wood-Dauphinee & Williams, 1987)	22	21	18	16	22	22	16	14	13	18	21	–	–
Beck depression inventory (short form)+ (Beck et al., 1974)	2	3	6	2	0	2	8	6	10	6	4	–	–
Quality of life (EQ-5D) (/100)(Oppe et al., 2007)	80	80	80	70	85	75	65	85	60	80	80	–	–

Note. Abbreviations*: Pt = Physiotherapist, OT = Occupational therapist, SLP = speech language pathologist, SW = Social worker, N = Nutritionist, Neuro = neuropsychologist, SEd = specialized educator; LFU = Lost to follow-up; R= right, L = left.

** Greater score indicates better reintegration to normal living.

+ 0–9: no or minimal depression; 10–18: mild to moderate depression; 19–29: moderate to severe depression; 30–39: severe depression.

### Affective Attitude

Most patients were satisfied with the intervention and emphasized the advantages of staying at home to get their rehabilitation treatments. This allowed them spare energy for their therapy. Most underlined the easiness of the platform and its suitability to their personal needs. No concerns emerged from the patients' side.

All therapists mentioned having a positive experience with the platform. Some even said that after the trial it would be missed in their daily interventions. The only concern expressed by a physical therapist was that he was stressed in the beginning of the project by the risk of fall: “I found it stressful, but we get used to it and give our instructions differently. I go further [at the rehabilitation center] in my exercises than at home because we cannot be nearby if he loses his balance, or something happens. I don't want [the patient] to injure his wife or himself.” (Physiotherapist, group 1)

### Burden

No patients mentioned obstacles that would lead them to ending their use of the platform. The learning curve was reported as very smooth, giving them enough time to adjust to the technology. When technical problems arose, participants underlined their appreciation of quick interventions from the research support team. Similar observations were made by therapists. A majority reported enjoying the opportunity to vary their schedule and the possibility to offer more frequent treatment sessions because they did not have to take account of patients' commuting, lifestyle, or fatigue. As one patient (#1, group 2) said: “You don't have to go anywhere. That, I think, is maybe the most important point for me. In my case, I have a wife who is disabled, and I can't leave her home alone, so it still allowed me to do some rehabilitation without leaving my home.” Both patients and therapists mentioned the need for on-demand access to the platform and specific technological equipment to be able to deliver all sorts of therapies without any quality compromises. This includes a computer screen for the therapists (more than 21 inches), a wider angle of vision for the camera to allow a better vision of the patient by the therapists, a portable arm for the tablet, and a document-camera for the patients, especially for speech therapies.

### Ethicality

None of the patients mentioned having experienced side effects from the treatments, namely headaches, dizziness, or fatigue. From the therapists' perspective, a neuropsychologist mentioned she was sometimes preoccupied by the level of confidentiality of her therapies since she could not know who was listening. The content of discussions with patients might contain information they do not wish to share with their spouse/caregiver. Alternative solutions were developed along the course of the project on a case-by-case basis. Clarifications were made when the presence of the spouse/caregiver was required.

When the lack of physical/social contact was brought up, there was no consensus whether it should be considered an advantage or a disadvantage of telerehabilitation. Upon recruitment, some patients reported being worried that by not being in a rehabilitation center they would be less supported, but then all reported that telerehabilitation provided the opposite. One patient felt “psychologically and physically supported.” Another patient said he preferred at home interventions because his disabilities were not exposed to everyone in the therapy room. This advantage was also brought up by therapists: “[The patients] see all kinds of disabilities when they come [to the gym]. Sometimes they might see a lot of other people walking around, amputees, this and that. Probably in their head, that's what they see. They're thinking, ‘Am I going to become like her?’ Or, ‘Look at him, he's not as bad.’ They don't necessarily always listen to you. But when you're in telerehabilitation, the client is really with you, focused. I can see the difference” (Physiotherapist, group 1).

On the other hand, some therapists were concerned because they feared their patients might be lacking social contact since they were not able share their problems with people who were going through the same situation. They estimated that lack of social contact could represent a disadvantage for people who were already isolated.

### Self-efficacy

Even though patients were intimidated by the technology at first, they all reported being confident about the use of the platform because it was simple to use (i.e., a large single green button appears when the tablet is turned on, and it is the only application available from the home page). The therapists took control of the rest of the session (for example, zooming in and out, and ending the session).

Therapist confidence to deliver perceived effective treatments was reported as being influenced by three different factors. First, a sense of helplessness was often described by some physical therapists who expressed the lack of tools to support themselves as therapists and to support the patient. A solution could take the form of a binder provided to the patient with illustrated exercises that will be asked of the patient along the course of the therapy. Second, most therapists mentioned the need to adapt and modify their treatments to provide high quality care. Some found this process difficult and were worried their therapy may be less effective than when delivered in-person. Third, there was a consensus between all therapists about the need to have a combination of telerehabilitation and in-person visits. There was a broad consensus on the need to provide the first evaluation in-person (e.g., 6 minutes walk test, dysarthria assessment, conversational therapy). As one suggested: “I found that telerehabilitation was very helpful, in the context that they were also coming [to the rehab center]. It was complementary because there are things that you don't see on screen. The quality of the movement, the control of the scapula, all that, is very difficult with the camera. But on the other hand, once you conduct your evaluation in person, it is super enriching and complementary” (Physiotherapist, group 2). Additionally, when required, patients might have to come for follow-up assessments. Some therapists emphasized the need to discuss this at the beginning of the therapy to set patients' expectations.

### Opportunity Cost

The biggest advantage for the patients was the time spared by not needing to commute. Therapists also mentioned that they had better interactions with the patients and found them to be more focused and motivated in their therapy: Sometimes we're one-on-one, yes, but [the patient] in the gym sees lots of other people walking around, amputees, this, that … He doesn't necessarily always listen to you; whereas when you are in telerehabilitation, the client is really with you, focused. I can tell the difference” (Physiotherapist, group 1).

### Intervention Coherence

Every patient understood the need to receive rehabilitation services after their stroke and it made sense for them to be treated via telerehabilitation. As one patient said: “The tablet, everyone should manage to use it. It motivated me and [the implementation of telerehabilitation] is very important, not only for patients with stroke, but for all kinds of other illnesses or health events requiring rehabilitation” (Participant 2). Moreover, therapists found that telerehabilitation was coherent with stroke rehabilitation because of the common need of high intensity and frequent care. They also found that telerehabilitation gave them an insight about their patients' personal environment that they never experienced before, including interactions between the patients and caregivers. A speech therapist said: “It struck me how, when I see them in real time, it's very rich - everything I'm going to look for in terms of communication, that I was losing with the individual sessions, that I didn't have as much” (Speech pathologist, group 2). However, therapists agreed that telerehabilitation was not suited for all types of treatments/patients. Indeed, some techniques (e.g., manual therapy, neurological rehabilitation, motor control, teaching of breathing pattern) or types of treatment (e.g., evaluation of functional activities in occupational therapy, communication therapy) were limited due to the virtual environment. In addition, the patient's profile was also to be considered. Patients with cognitive impairments, speech and language impairments, or musculoskeletal pain require different interventions and management was likely less suitable.

### Perceived Effectiveness

Every patient perceived improvement in their function and reported reaching their personal goals. Therapists were more skeptical in the beginning because of some technical problems or their feelings of not knowing how to organize their therapy. With time, they had learned to be more confident with the platform and perceived that overall, it was effective with this population: “What should you do? How should you conduct an evaluation? You're so unused to this. You're a little confused, especially the first one. But after that, you gain confidence, and on the second you try something else, and on the third you try something else. And it's no longer unknown” (OT, group 1). With the platform, therapists believed that they could promptly suggest modifications to the patient and be assured that they would do their exercises correctly during unsupervised sessions at home. In addition, when patients recognized their capability to perform their program adequately it improved their adherence and self-esteem, leading in turn to more effective therapy. As one occupational therapist said: “One of the very positive points is that since we use the patient's home equipment, we really use the home as a practice environment, the client is more likely to exercise between sessions. He feels comfortable doing them, he feels confident” (Physiotherapist, group 2).

## Discussion

Telerehabilitation platforms for the delivery of rehabilitation therapies in a stroke continuum of care are getting attention. In this study, we explored the acceptability of using such platforms with both patients and multidisciplinary rehabilitation therapists and found overall good acceptability, based upon a theoretical framework to operationalize the concept thoroughly. Nonetheless, different nuances emerged between user groups, with therapists expressing concerns with respect to patient safety, the need to provide patients with complementary tools to facilitate the conduct of the recommended exercises remotely, and the need to combine in-person and remote sessions.

This study confirms the acceptability of telerehabilitation and allows us to identify from a therapist's perspective, ways to improve the delivery of sessions in a population with stroke. To smooth the transition from in-person interventions to telerehabilitation, therapists need to be supported and guided throughout the process (Brouns et al., 2018). It is important to underscore that telerehabilitation should not be seen as simply providing in-person rehabilitation in front of a screen. Thus, we recommend that therapists receive continued training to help them to adapt their therapies. As telerehabilitation platforms evolve rapidly, a community of practice could also be beneficial to share knowledge and experience with peers. Moreover, special consideration must be given to platform access, as it needs to be always available with no restrictions on the number of concurrent users. This would allow all therapists to work simultaneously, without having to sacrifice therapy time to adapt to other team members' schedules.

In contrast with what others reported previously (Brouns et al., 2018; Davoody & Hagglund, 2016; Tyagi et al., 2018), once the learning curve was reached, therapists in this study did not report the need to plan for extra time to account for a remote delivery of care.

Recommendations also arose from patients' perspectives to improve the acceptability of a telerehabilitation platform. In this study, we provided all the equipment necessary to both patients and therapists including a good quality Internet connection. This was crucial for smooth delivery of the intervention, especially since just-in-time cues are important for optimal conduct of physical therapy sessions. The delivery of the therapy via our platform was found not optimal through smartphones, and therefore such patient devices were not allowed. Equipment requirements are to be considered to avoid inequity of care for patients who would not have access to those resources (Bienassis et al., 2020; Hashiguchi, 2020; Rich et al., 2019; Veinot et al., 2018). Rich et al. (2019) showed that an inequality gap could remain despite increasing access to adequate equipment as low familiarity with technology remains a barrier. This reinforces the need to develop user-friendly platform interfaces.

Previous authors have brought up concerns about the need for elderly patients to adapt to the new technology (Jaffe et al., 2020; Tyagi et al., 2018). In this study, lack of familiarity with technical devices was not found to be a barrier to the acceptability of the platform. We circumvented this challenge by providing users with constant support and minimizing visual cues and commands on the patient platform's interface. A starting kit including basic stroke rehabilitation gear such as mini bands, small weights, and illustrated exercises could also favor the uptake of the platform from a patient's perspective and limit the perceived gaps between in-person and remote modes of delivery.

The lack of social contact due to remote therapy was observed by some therapists, especially for patients who did not benefit from an adequate social network. This was not echoed by patients. In general, patients saw advantages to not having to commute for their therapy. Most mentioned that telerehabilitation freed time up to engage with people from the community (e.g., social activities, activities of daily living), whereas most of their energy would have been spent on commuting in a traditional in-person model of rehabilitation. This is similar to other studies of telerehabilitation that concluded that patients felt less isolated (Crotty et al., 2014), more connected because they had quality time with their therapist (Chen et al., 2020), safer and more confident (Hoaas et al., 2016).

### Study Limitations

We cannot assure that the results of this study are transferable to any stroke telerehabilitation users since the platform used is pivotal to the acceptability of the intervention. The platform used in this study was developed to be user friendly to avoid difficulties for participants with low technological literacy. The research environment surrounding the trial of the platform, (i.e., providing the equipment at no cost for the participants), could have limited the expression of barriers, especially for the ethicality and the opportunity cost components studied. Participants were invited by the research team members who conducted the interviews to share any thoughts, positive or negative, about the use of the platform. However, social desirability bias cannot be ruled out. Further investigation in terms of case mix (e.g., type of stroke, rehabilitation needs) and caregiver perspective is warranted.

## Conclusions

This study is one of the first to explore in-depth the acceptability of a telerehabilitation platform for stroke rehabilitation beyond patient and therapist satisfaction. Sekhon et al.'s (2017) conceptual framework proved useful to operationalize and compare the perceptions of both types of users. It allowed us to identify factors that must be considered for implementing a telerehabilitation intervention and provide recommendations for a smooth and efficient use of this technology. The study confirms the need to use a platform which requires a minimum of user manipulation. It also raises issues of social isolation and the need for therapists to rethink how therapies are delivered in a virtual environment.

## Appendix Excerpts from the Interview Guides

1. What was your experience with this telerehabilitation intervention?
2. How were the interactions with the health care professionals/patients in this intervention?
3. Before you started using the platform, how did you feel about your capacity to use this kind of technology?
4. How did you experience the « getting to know" part of the platform?
5. Could you tell me about your fears related to the use of the telerehabilitation platform?
6. Did the fears you had in the beginning turned out to be true while using the platform?
7. Could you talk to me about the efforts you had to put in to use the telerehabilitation platform?
8. Would you say you had to give up something to take part in this intervention?
9. Could you tell me what you think about the drawbacks and advantages related to your participation to this intervention?
10. Would you say the intervention fulfilled your expectations?
11. Could you give me an example of objectives you had prior to the beginning of the intervention that was not fulfilled by the telerehabilitation platform?
